# 925 Reduction of Catheter Associated Urinary Tract Infections with Introduction of New Collection Device

**DOI:** 10.1093/jbcr/iraf019.456

**Published:** 2025-04-01

**Authors:** Natalie Fitzgerald, Shawna Thomas, Brett Hartman

**Affiliations:** Richard M. Fairbanks Burn Center; Eskenazi Health; Eskenazi Health

## Abstract

**Introduction:**

In 2022 the Richard M. Fairbanks Burn Center adopted the utilization of a new foley catheter and collection system. This system was brought in to help the team monitor urine output more accurately. However, a secondary benefit has been noticed since it’s adoption.

**Methods:**

The Accuryn urine collection system was adopted for utilization in all fluid resuscitations that were admitted to the burn center at the start of 2021. The initial plan for adoption was only to see its impact on urine output documentation. The system also provided the ability to continuously monitor temperatures without additional components and obtain abdominal compartment measures if indicated. No other changes were made in our process with regards to placement, anchoring or removal of foley catheters.

**Results:**

In 2020 the burn center had 4 catheter associated urinary tract infections (CAUTI) and in 2021 there were 3. These infections were seen even with the utilization of a bundle practice for foley catheter care and utilization. Since the implementation of the Accuryn catheter and monitoring system in 2022 the burn center had no CAUTIs in 2022 and 2023. There was 1 CAUTI in 2024 but of note that was seen in a patient with a standard foley catheter.

**Conclusions:**

This catheter did not only provide assistance with the evaluation of urinary output but also the reduction of CAUTIs within this center.

**Applicability of Research to Practice:**

Further investigation is needed regarding if these results are transferable to other burn centers or even critical care units. These catheters have proved beneficial for this center in regard to infection reduction and utilization will be continued. Some might even question if the cost benefit of the catheter. However, any infection in the immunocompromised burn patient is important to avoid and this device has proven to be beneficial in this regard.

**Funding for the Study:**

No funding was received for this study.

